# Contraceptive care and method use by sexual and gender minority status: insights from a longitudinal panel of sexual and reproductive healthcare patients in Wisconsin

**DOI:** 10.1080/26410397.2025.2544432

**Published:** 2025-08-11

**Authors:** Ellie Leong, Christina E. Geddes, Fiona Weeks, Megan L. Kavanaugh

**Affiliations:** a Senior Research Associate, Guttmacher Institute, New York, NY, USA. *Correspondence*: eleong@guttmacher.org; b Senior Research Assistant, formerly of Guttmacher Institute, New York, NY, USA; c Researcher, University of Wisconsin School of Medicine and Public Health, Madison, WI, USA; d Principal Research Scientist, Guttmacher Institute, New York, NY, USA

**Keywords:** contraception, contraceptive access, preferred contraceptive use, barriers to care, inequities in health care

## Abstract

Access to sexual and reproductive health (SRH) care is key for people to realise their reproductive goals, but sexual and gender minority (SGM) patients may experience different barriers or facilitators to access than their non-SGM counterparts. We analysed a panel dataset of 900 patients using publicly funded SRH services in Wisconsin in 2020–2023 and constructed conditional logistic regression models to explore barriers to contraceptive services and subsequent patient-centred contraceptive method use, stratified by SGM status. Experience of barriers to wanted contraception was strongly related to preferred contraceptive use (aOR = 0.36, CI: 0.20–0.64, *p* = 0.002) and method satisfaction (aOR = 0.39, CI: 0.20–0.77, *p* = 0.010). Barriers were also linked to lower use of LARC methods by patients preferring LARC methods, including affordability barriers (aOR = 0.09, CI: 0.01–0.85, *p* = 0.037). SGM patients were less likely to report preferred method use (aOR = 0.18, CI: 0.08–0.42, *p* = 0.001) and method satisfaction (aOR = 0.30, CI: 0.11–0.81, *p* = 0.022) after experiencing barriers. Those who experienced affordability barriers were also less likely to report preferred method use (aOR = 0.18, CI: 0.05–0.68, *p* = 0.015). For non-SGM patients, only experiencing a missed healthcare visit was related to lower method satisfaction (aOR = 0.48, CI: 0.25–0.92, *p* = 0.029). Our study highlights that barriers to contraceptive care can hamper people’s ability to realise their contraceptive preferences. Furthermore, our differential findings by SGM status point to potential gaps in healthcare systems that are not adequately set up to serve all patients.

## Plain language summary

Barriers to accessing contraceptive care can make it difficult for people to fulfil their sexual and reproductive health (SRH) desires. These barriers can affect people differently based on their identities, like gender and sexual orientation. Our analysis explored barriers individuals face when trying to access contraceptive methods and how those might affect their ability to use the method they want or how they feel about their method. We paid special attention to individuals who identify as sexual and gender minority (SGM). We used data from a study of SRH patients in Wisconsin who visited publicly funded clinics in 2020. The study followed these patients every 6 months for 2 years. The data describe their experiences accessing SRH care and using contraceptive methods. We found that those who experienced barriers to care were less likely to be using the method they wanted, especially if they wanted to use long-acting reversible methods, and were less likely to be satisfied with them. We examined these relationships for SGM and non-SGM respondents separately. For SGM respondents, experience of barriers was linked to reduced preferred contraceptive use and method satisfaction. For non-SGM respondents, only missing a healthcare visit reduced method satisfaction. These findings highlight the need to redesign and rebuild systems of contraceptive care delivery. These systems need to centre the needs of individuals who may be particularly impacted by barriers to care, such as SGM individuals. Expanding protective reproductive health policies at both state and federal levels could reduce patient access barriers and improve patient-centred outcomes.

## Résumé

L’accès aux soins de santé sexuelle et reproductive (SSR) est essentiel pour atteindre ses objectifs reproductifs, mais les patients issus de minorités sexuelles et de genre peuvent rencontrer des facteurs entravant ou facilitant l’accès qui diffèrent de ceux rencontrés par leurs homologues n’appartenant pas à des minorités. Nous avons analysé un ensemble de données de 900 patients qui avaient utilisé des services de SSR financés par des fonds publics dans le Wisconsin entre 2020 et 2023 et nous avons construit des modèles de régression logistique conditionnelle afin d’étudier les obstacles à l’accès aux services de contraception et l’utilisation ultérieure de méthodes contraceptives centrées sur le patient, stratifiés selon l’appartenance ou non à une minorité sexuelle et de genre. L’expérience des obstacles à la contraception souhaitée était fortement liée à l’utilisation de la contraception préférée (RCa = 0,36, IC: 0,20-0,64, *p* = 0,002) et à la satisfaction quant à la méthode (RCa = 0,39, IC: 0,20-0,77, *p* = 0,010). Les obstacles étaient également liés à une utilisation moindre des méthodes LARC (contraception réversible à longue durée d'action) par les patients préférant ces méthodes, y compris les obstacles liés à l’accessibilité financière (RCa = 0,09, IC:0,01-0,85, *p* = 0,037). Les patients issus de minorités sexuelles et de genre étaient moins susceptibles de déclarer l’utilisation de leur méthode préférée (RCa = 0,18, IC: 0,08-0,42, *p* = 0,001) et la satisfaction quant à la méthode (RCa = 0,30, IC: 0,11-0,81, *p* = 0,022) après avoir rencontré des obstacles. Ceux qui avaient rencontré des obstacles liés à l’accessibilité financière étaient également moins susceptibles de déclarer l’utilisation de la méthode préférée (RCa = 0,18, IC: 0,05-0,68, *p* = 0,015). Pour les patients n’appartenant pas à une minorité sexuelle ou de genre, seule une visite manquée au centre de soins était liée à une satisfaction moindre à l’égard de la méthode (RCa = 0,48, IC: 0,25-0,92, *p* = 0,029). Notre étude souligne que les obstacles à la contraception peuvent entraver la réalisation des préférences en matière de contraception. De plus, nos résultats différenciés selon le statut d’appartenance à une minorité sexuelle ou de genre montrent des lacunes potentielles dans les systèmes de santé, qui ne sont pas organisés de manière à desservir tous les patients.

## Resumen

El acceso a los servicios de salud sexual y reproductiva (SSR) es clave para que las personas puedan realizar sus objetivos reproductivos, pero los pacientes de minorías sexuales y de género (MSG) podrían enfrentar diferentes barreras o facilitadores al acceso que sus homólogos no de MSG. Analizamos un conjunto de datos de panel de 900 pacientes que utilizaron servicios de SSR financiados con fondos públicos en Wisconsin, en 2020-2023, y creamos modelos de regresión logística condicional para explorar las barreras a los servicios de anticoncepción y al uso subsiguiente de métodos anticonceptivos centrados en los pacientes, estratificados por estado de MSG. La experiencia de barreras a la anticoncepción deseada estaba relacionada en gran medida con el uso del anticonceptivo preferido (RMa = 0.36, IC: 0.20-0.64, *p* = 0.002) y la satisfacción con el método (RMa = 0.39, IC: 0.20-0.77, *p* = 0.010). Además, las barreras estaban vinculadas con menor uso de métodos LARC por pacientes que preferían métodos LARC, tales como barreras relacionadas con la asequibilidad (RMa = 0.09, IC: 0.01-0.85, *p* = 0.037). Los pacientes de MSG eran menos propensos a informar uso de su método preferido (RMa = 0.18, IC: 0.08-0.42, *p* = 0.001) y satisfacción con el método (RMa = 0.30, IC: 0.11-0.81, *p* = 0.022) tras enfrentar barreras. Las personas que enfrentaron barreras de asequibilidad también eran menos propensas a informar uso de su método preferido (RMa = 0.18, IC: 0.05-0.68, *p* = 0.015). En el caso de pacientes no de MSG, faltar a una consulta médica fue el único factor relacionado con una menor satisfacción con el método (RMa = 0.48, IC: 0.25-0.92, *p* = 0.029). Nuestro estudio destaca que las barreras a los servicios de anticoncepción pueden obstaculizar la capacidad de las personas para realizar sus preferencias anticonceptivas. Además, nuestros hallazgos diferenciales por estado de MSG indican posibles brechas en los sistemas de salud que no están preparados adecuadamente para atender a todo tipo de paciente.

## Introduction

People use contraceptive methods for diverse and often multiple reasons to realise their sexual and reproductive health (SRH) needs^1,2^ as well as general health and well-being.^3^ Two key aspects of patient-centred access to care include the **availability** of services (proximity to care, accommodation of needs, hours of operation, and flexible mechanisms for scheduling) and **affordability** of care (direct service costs, indirect costs such as transportation and childcare, and opportunity costs such as wage loss).^4^ Previous research has elucidated barriers to both affordability^5,6^ and availability^7^ of care in the United States. These barriers hinder people interested in contraception from obtaining their preferred method or sometimes any contraception at all,^8–10^ impacting people’s ability to fulfil their desired SRH outcomes.^11,12^

Moreover, there is evidence that barriers to contraceptive care are higher among people who belong to marginalised communities.^5,6,8,13^ People who identify as sexual and gender minorities (SGM),* including lesbian, gay, bisexual, transgender, queer, and non-binary individuals, may be uniquely affected by SRH care barriers. A lack of healthcare provider competency in working with SGM-identifying patients or overt anti-SGM discrimination can lead to delays in care and treatment through the unavailability of appropriate care.^14,15^ Additionally, SGM individuals are more likely than their non-SGM peers to be uninsured,^16^ which affects the affordability of care.

Federal policies have a significant influence on SRH care across all states. However, state-level policies also wield considerable power in determining the accessibility of contraceptive services. Previous studies have found that state-level disruptions in public funds at SRH care sites increased costs for contraceptive methods and disrupted patients’ ability to access care.^17,18^ In Wisconsin, these compounding barriers have led to delayed care and lower quality care, particularly affecting SGM individuals.^19^

Previous research studying utilisation of and barriers to contraceptive care largely relies on cross-sectional data.^6,20,21^ In this study, we leverage longitudinal data from a panel of patients who accessed SRH care at publicly funded sites in Wisconsin in 2020 and reported on their SRH care-seeking behaviour and outcomes over a two-year period. The longitudinal panel data allow us to observe differences in the experience of care on subsequent SRH outcomes, contextualised within the individual. We explore differences in experiences of barriers to care and their association with contraceptive method use outcomes and assess differences in these experiences by SGM status.

## Methods

We analysed longitudinal data collected between February 2020 and June 2023, pausing briefly between March and July 2020 due to the emergence of the COVID-19 pandemic. These data were collected using the Wisconsin Family Planning Clinic Patient Survey as a component of the Reproductive Health Impact Study (RHIS), an extensive multi-state study conducted by the Guttmacher Institute, which aimed to document the impact policy changes have had on the publicly funded SRH care system. NORC at the University of Chicago managed data collection.

### Respondents

Facilities were sampled into the study based on the following criteria: (1) received any federal /or state public funds to support SRH care services at the start of baseline fielding in years 2020–2021;^†^ (2) served 200 + patients assigned female at birth annually; and (3) open as of December 2019. Out of 60 eligible sites available and recruited, 21 agreed to participate and completed baseline fielding by successfully collecting a target number of responses based on patients served during their data collection period. Patients were invited to participate if they were 15 years of age and older, assigned female at birth,^‡^^§^ and sought SRH care** at a sampled publicly funded healthcare facility. An overall target sample size of 2200 respondents at baseline was calculated by NORC to have sufficient power to detect changes in contraceptive method use at the end of longitudinal data collection, with an anticipated 55% opt-in rate into the longitudinal panel. The average baseline response rate by care-seeking individuals across participating clinics was 33% with a total baseline sample of 2,375 eligible patients who completed the survey and a final 1653 respondents who opted in to longitudinal follow-up. Additional detail on sampling and data collection protocols for the baseline survey, as well as the multi-stage weighting procedure implemented for the baseline sample to reflect patients served at the universe of publicly funded clinics in Wisconsin in 2020, has been described elsewhere.^20,22^

Patients who consented to follow-up surveys at baseline were contacted every 6 months for 24 months between August 2020 and June 2023. Of the 1653 respondents who opted in to the longitudinal follow-up, 703 completed the follow-up survey at 6 months, 768 completed at 12 months, 758 completed at 18 months, and 799 completed at 24 months. Baseline and follow-up surveys were available in English and Spanish.

As the focus of our analysis was on contraceptive outcomes, we narrowed our analytic sample to respondents who did not report trying to become pregnant at any study timepoint. We additionally limited respondents to those who completed the baseline survey and completed at least one follow-up survey. Of the 2375 completed responses at baseline, we dropped 25 respondents who reported being pregnant (unconfirmed with test), 1352 respondents who only responded to the baseline survey and 98 respondents who reported wanting to become pregnant at any study time point. Our final analytic sample included 3607 responses from 900 unique respondents.

### Variables

#### Sexual and gender minority status

Gender identity was assessed using a two-step measure that asks about sex assigned at birth separately from current gender.^23^ We then constructed a binary SGM group variable based on responses to the questions on gender identity and sexual orientation. For gender identity, respondents were asked broadly “How do you describe yourself?” and were able to select multiple options from the following: “woman”, “man”, “transgender”, “non-binary”, “something else” with the option to write in a response, “don’t know”, and “prefer not to answer.” For sexual orientation, respondents were asked “Which of the following best represents how you think about yourself?” and were able to select multiple options from the following: “lesbian or gay”, “straight”, “bisexual”, “pansexual”, “queer”, “something else” with the option to write in a response, “don’t know”, and “prefer not to answer.” We considered respondents who only reported being a woman and straight as “cisgender and heterosexual”. Respondents who selected any other category, including 36 respondents who selected “don’t know” to either question were categorised as “transgender and/or queer”. Respondents who selected “prefer not to answer” to both questions (n = 36) were not included in any SGM status-specific analysis. As identities are fluid, respondents were asked questions about how they identify at each time point, and we considered all responses as valid. However, we made an adjustment to the SGM definition for the stratification used in the conditional logistic regression models to include respondents who identified as SGM at any timepoint as part of the SGM group. This allowed us to include responses for as many survey timepoints as a respondent completed, even as their identity shifted. This binary SGM variable combined gender identity and sexual orientation to maximise sample sizes for analysis. However, the SRH care needs and experiences of sexual minority and gender minority individuals are unique. We discuss evidence from prior studies and implications in the discussion.

#### Barriers to contraceptive care

We conceptualised potential barriers and disruptions to care in four ways: general access barriers, measured as missing any needed healthcare visit and trouble getting contraceptive care for any reason, and then specifically reported reasons for trouble getting care due to availability or affordability. For a missed healthcare visit, respondents were asked if over the previous 12 months (baseline) or 6 months (biannual) there was a time when they wanted healthcare for any reason but didn’t get it (yes/no/prefer not to answer)*.* At each survey timepoint, respondents were asked if over the previous 12 months (baseline) or 6 months (biannual) they had “delayed or had trouble getting their wanted contraceptive method” (yes/no/prefer not to answer).

If respondents reported a delay or trouble getting their wanted contraceptive method, they were asked to report the reason why they experienced the delay. We considered respondents who reported any of the following reasons as experiencing an availability-related delay: “it was too difficult to get to (no transportation or childcare, couldn't take time off work)”,^††^ “the method that I wanted was not available at my doctor's office, clinic or pharmacy”, “my source of health care is religiously affiliated, and thus does not provide the birth control method I wanted”, and “the doctor's office, clinic or pharmacy wasn't open when I could get there.” We categorised respondents who reported any of the following reasons as experiencing an affordability-related barrier: “I couldn’t afford it”, “I don’t have insurance”, “my health insurance doesn’t cover it”, “the insurance co-pays/deductibles were too high.”

#### Patient-centred contraceptive method use outcomes

We conceptualised patient-centred contraceptive method use outcomes in five ways: use of preferred method, satisfaction with current method, and fulfilment of a long-acting reversible contraceptive (LARC), short-acting reversible contraceptive (SARC), and coital methods. Fulfillment in this context refers to the proportion of respondents who reported successfully using a method type they reported wanting to use.

All respondents were asked what contraceptive method they would use if they could use any. Response options included a grid of contraceptive methods (yes/no/prefer not to answer) as well as a response option prior to the grid response options for respondents to indicate if they were currently using their preferred method.

Respondents who answered “yes” to contraceptive use in the last three months were asked a series of contraceptive use questions, including their level of current method satisfaction using a 5-point rating scale ranging from “very satisfied” to “very dissatisfied”. If respondents indicated that they used more than one method, they were asked to report their satisfaction with the method they used most often. Respondents who reported being “very satisfied” or “satisfied” were categorised as satisfied with their method, and all other respondents, including the neutral category “neither satisfied nor dissatisfied”, were considered not satisfied with their method.

We constructed fulfilment by method type from two survey items: current use and preferred method use. For each possible method, respondents who reported a preference for a specific method and reported using that method were considered fulfilled. We then grouped by method type: LARC (IUD, implant); SARC (Depo Provera, oral contraceptive, patch, ring); permanent (tubal ligation, partner’s vasectomy); and coital (condom, barrier, natural family planning, emergency contraceptive, withdrawal). Individuals who would be considered meeting fulfilment for a particular method within a grouping are considered fulfilled for that method group.

#### Covariates

We included time-varying socio-demographic characteristics as covariates in our multivariate models: relationship status, employment, residence type, and insurance. We used a dichotomous relationship variable representing those who were married and/or cohabiting and those who were neither married nor cohabiting. We included a dichotomous employment status variable, which included respondents who were employed (including self-employed), and all other employment statuses (student, out of work for one year or more, out of work for one year or less, a homemaker, retired, or unable to work) were categorised as not employed. We categorised respondents as residing within rural or urban residences according to the United States Health Resources and Services Administration’s definition,^24^ matching respondent zip codes to RUCA codes, a resource^25^ from the United States Department of Agriculture (USDA). Respondents were able to select as many types of coverage as possible and those who reported BadgerCare Medicaid or Family Planning Only Services, Medicare, TRICARE or other military healthcare, Indian Health Service, insurance through a current or former employer, union, school, insurance purchased directly from the marketplace, or any other eligible written in response were coded as insured. Across all analytic variables, respondents who reported “prefer not to answer” were set to missing for the corresponding item.

### Analytic strategy

We first conducted descriptive analyses of our weighted analytic sample, both overall and by SGM status. We ran simple logistic regressions to examine differences in socio-demographic characteristics at baseline by SGM and Chi-Squared tests of independence for both the overall sample and stratified by SGM to examine differences in time trends. We constructed conditional logistic regression models to examine associations between barriers to care (e.g. any trouble getting wanted contraceptive method, trouble due to availability, trouble due to cost, and missed healthcare visit) and contraceptive method use outcome (e.g. use of preferred method, method satisfaction, and contraceptive fulfilment of LARCs, SARCs, and coital methods). We were unable to examine fulfilment of permanent methods as an outcome in the models due to limited observations. The models controlled for all time-invariant characteristics within respondents to examine respondent-level fixed effects related to changes in independent variables. These models also accounted for the hierarchical nature and sampling design of these data. All models utilised panel weights constructed from the baseline sample weights designed to represent the publicly funded female SRH care patient population over the age of 15 in Wisconsin.

Full analytic sample models were adjusted for the following time-varying socio-demographic characteristics known to be associated with contraceptive outcomes: relationship status, employment, residence location, and insurance status. We additionally provide estimates stratified by sexual and gender minority status to examine if the relationship between barriers to care and subsequent patient-centred contraceptive method use outcomes varies by one’s sexual and gender identity. In the models stratified by SGM, we only included relationship status, employment, and residence location due to the limited number of SGM respondents in the uninsured group. We also focused on the broader outcomes of current use of a preferred method and satisfaction with method use in the models stratified by SGM, as we were limited in the number of observations to examine fulfilment by method type.

Overall, we observed less than 6% missing respondents (n = 51) for any covariates included in the models. When we additionally considered missing respondents in the variables of interest, we observed up to 77 respondents (9%) missing. We utilised listwise deletion in our analyses as there were no substantial differences in observable characteristics between these respondents and respondents retained in the models.

We performed sensitivity analyses focusing on the demographic composition of our analytic sample and found no differences in socio-demographic characteristics between the original full sample and our analytic sample. Additionally, we did not find differences in characteristics or model results if we removed the 36 respondents who reported “don’t know” for either gender or sexual orientation. Unadjusted and adjusted odds ratios and 95% confidence intervals are presented from the models. All analyses were performed in Stata 18.0.

### Informed consent and IRB approval

Study protocols for the Wisconsin Family Planning Clinic Patient Survey were approved by both the NORC Institutional Review Board (DHHS identifier IRB00000967) from 2 February 2018, to 19 July 2023 and the Guttmacher Institute Institutional Review Board (DHHS identifier IRB00002197) from 25 January 2018 to 30 September 2024. All study participants provided verbal consent to receive information about the baseline study prior to receiving the survey from clinic staff and additionally indicated their consent electronically after receiving a written introduction to participant rights. Respondents consented to follow-up surveys at baseline by indicating that they would like to be contacted for follow-up surveys and providing their contact information. For the inclusion of adolescent participants 15–17 in this study, a request to conduct research involving minors and a Waiver of Documentation of Consent negating the need for parental written consent for minors to participate was submitted. This request and waiver were granted because (1) the research involved no more than minimal risk to the subjects, (2) the waiver or alteration did not adversely affect the right and welfare of the subjects, (3) the research could not practically be carried without the waiver or alteration, and (4) whenever appropriate, the subjects would be provided with additional pertinent information after participation.

## Results

In Table 1, we present baseline demographic characteristics of the 900 respondents in our analytic sample, which includes 651 cisgender and heterosexual respondents (72%) and 239 transgender and/or queer respondents (28%). We did not find any differences in baseline demographic characteristics by SGM. Respondents were on average 27 years old, with over 61% indicating incomes at or above 200% of the federal poverty level. More than 62% of respondents had at least some college education or held an associate's degree. A small fraction of respondents (<5%) reported being born outside the United States. Most respondents (61%) identified as non-Hispanic White, and more than 15% identified as non-Hispanic Black. Approximately 63% stated that they were neither married nor cohabiting. Nearly two-thirds of respondents (66%) were employed for wages, and an additional 5% are self-employed. A substantial majority, 92%, had some type of insurance, and 80% reported residing in an urban zip code.

**Table 1.** Demographic characteristics of Wisconsin sexual and reproductive health patients ages 15+ at baseline not trying to become pregnant, 2020

	Baseline sample		Cisgender and heterosexual respondents		Transgender and/or Queer respondents		Diff. at baseline by sexual and gender minority status (*p*-value)
*Respondents not trying to become pregnant (Ns)*	900		651		239		
**Age^a^**							
Mean years (standard deviation)	27.22 (8.00)		27.91 (8.73)		25.44 (5.52)		
15–17	4.23	32	5.34	27	1.51	5	0.051
18–19	11.99	95	10.98	59	14.41	33	
20–24	27.73	282	24.87	187	35.38	94	
25–29	23.65	232	22.68	166	26.66	64	
30–34	15.99	126	15.44	96	16.10	26	
35–39	8.10	65	10.17	58	3.03	7	
40–44	5.70	46	7.03	37	2.45	9	
45+	2.62	22	3.49	21	0.46	1	
**Income as a % of the federal poverty level^a^**							
<100%	18.32	297	19.22	219	15.32	71	0.571
100–199%	20.60	295	21.20	217	19.44	77	
200%+	61.07	308	59.58	215	65.24	91	
**Educational attainment^a^**							
Less than high school	6.67	52	7.71	41	4.06	10	0.260
High school graduate or equivalent	30.86	295	31.17	207	29.37	82	
Some college or Associates	36.52	347	35.65	251	39.92	96	
College graduate or more	25.95	201	25.47	149	26.65	50	
**Born outside the United States**	4.26	40	3.90	26	4.74	11	0.707
**Race and ethnicity**							
White, non-Hispanic	61.25	507	62.99	368	57.87	138	0.832
Black, non-Hispanic	15.20	156	15.90	125	13.74	31	
Asian, non-Hispanic	1.73	17	2.15	14	0.37	2	
Native American, Alaska Native, American Indian or Native Hawaiian or Pacific Islander, non-Hispanic	1.47	7	1.24	4	2.10	3	
Multiple races, non-Hispanic	7.73	67	6.62	45	10.06	21	
Hispanic/Latina/x	12.62	141	11.10	93	15.86	43	
**Gender identity and sexual orientation^b^**							
Cisgender and heterosexual	72.06	651	–	**–**	**–**	**–**	
Transgender and/or queer	27.94	239	**–**	**–**	**–**	**–**	
**Gender identity**							
Cisgender	97.36	879	**–**	**–**	**–**	**–**	
Transgender	0.38	3	**–**	**–**	**–**	**–**	
Other gender identity (including non-binary)	2.26	16	**–**	**–**	**–**	**–**	
**Sexual orientation**							
Lesbian or gay	0.88	5	**–**	**–**	**–**	**–**	
Straight	72.73	653	**–**	**–**	**–**	**–**	
Bisexual	17.09	160	**–**	**–**	**–**	**–**	
Pansexual	4.93	38	**–**	**–**	**–**	**–**	
Something else	0.35	2	**–**	**–**	**–**	**–**	
Queer	3.47	25	**–**	**–**	**–**	**–**	
Asexuality spectrum	0.54	3	**–**	**–**	**–**	**–**	
**Relationship status^b^**							
Married	11.73	83	12.28	62	9.89	18	0.854
Cohabiting	25.22	224	27.10	169	20.25	53	
Neither married nor cohabiting	63.05	582	60.62	413	69.86	167	
**Employment^b^**							
Employed for wages	65.90	562	64.93	407	69.08	153	0.075
Self-employed	5.39	46	6.27	38	2.54	7	
Student	16.77	159	17.91	117	13.90	40	
Unemployed	7.62	77	6.26	53	11.41	24	
A homemaker	2.08	20	2.16	12	1.34	5	
Retired or unable to work	2.24	27	2.46	20	1.73	7	
**Health Insurance Coverage^b^**							
No insurance	8.47	67	8.21	50	8.93	16	0.890
Private insurance	46.24	332	44.42	234	51.82	98	
Public insurance	40.17	434	41.66	321	35.78	109	
Other insurance	5.12	46	5.71	35	3.47	10	
**Location**							
Urban	79.82	749	77.40	534	85.43	205	0.070
Rural	20.18	136	22.60	107	14.57	29	

Notes: Presented are unweighted sample N and weighted proportions. Respondents were included in the analysis if they received sexual and reproductive health care at baseline from a publicly supported health care centre that served 200 or more sexual and reproductive health patients in Wisconsin in 2020, if their sex assigned at birth was female, if they did not report a pregnancy at baseline, if they did not report trying to become pregnant at every survey time point, and if they completed the baseline survey and at least one follow-up survey at 6, 12, 18, or 24 months. Some characteristics do not sum to 100% due to rounding. ^a^Only asked at baseline. ^b^These variables are time-varying and may have changed across the survey time periods.

**Figure 1.**
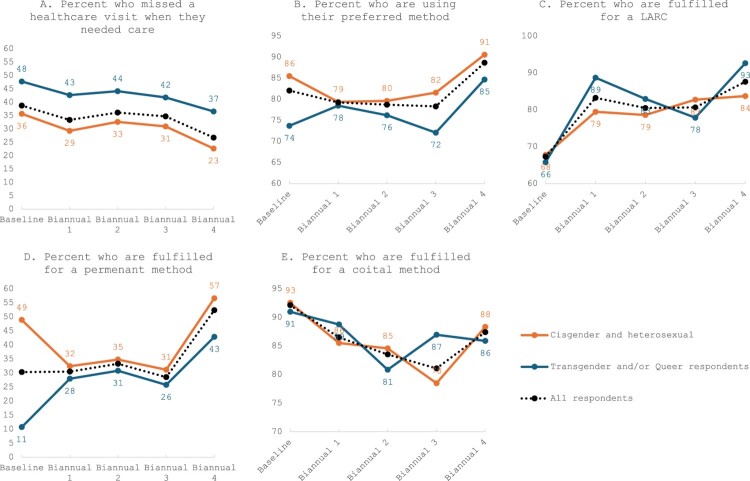
Differential changes in access to care and use of a preferred method or method type in Wisconsin, by sexual and gender minority status, 2020–2023

Full tabulations of every key indicator are available in Appendix 1, and these time trends were examined to contextualise the results of the models. We highlight selected key differences in trends of barriers to care and patient-centred outcomes over time, within the overall sample and within each subgroup in Figure 1, specifically trends that differ between SGM respondents and non-SGM respondents or present a consistent gap in outcomes between the two groups. While proportions of cisgender and heterosexual respondents who indicated missing a healthcare visit in the past 12 or 6 months decreased over time from 36% to 23% (*p* = 0.003), the proportion of transgender and/or queer respondents who reported missing a healthcare visit declined to a smaller extent from 48% to 37% (*p* = 0.449) and remained higher across all time periods. Reported use of a preferred method also increased over time among cisgender and heterosexual respondents, first dipping from 86% to 79% and then eventually rising to 91% (*p* = 0.004). In contrast, use of a preferred method remained relatively stable across the first 18 months, around 72–78% and rising at 24 months to 85% for transgender and/or queer respondents (*p* = 0.224) and remained consistently lower than cisgender and heterosexual respondents. When examining fulfilment by method type, differences emerged over time for LARC, permanent, and coital methods. Fulfilment of LARC method increased rapidly across the time periods among transgender and/or queer respondents, rising from 66% to 93% (*p* = 0.002), and fulfilment of permanent methods increased from 11% to 43% (*p* = 0.048). Meanwhile, among cisgender and heterosexual respondents, fulfilment of LARC methods also increased but to a lesser extent across the time periods from 68% to 84% (*p* = 0.107), and fulfilment of permanent methods decreased first from 49% to around 31–35% before rising again to 57% (*p* = 0.113). Fulfilment of coital methods, however, decreased among both cisgender and heterosexual respondents and transgender and/or queer respondents, dropping from 93% to 88% (*p* = 0.003) and from 91% to 86% (*p* = 0.454) respectively.

In Table 2, we present unadjusted and adjusted odds ratios for the relationship between barriers to care and patient-centred contraceptive method use outcomes. After adjusting for time-varying covariates, experiencing trouble getting wanted contraceptive method was associated with lower odds of currently using a preferred method (aOR = 0.36, CI: 0.20–0.64, *p* = 0.002), satisfaction with current method (aOR = 0.39, CI: 0.20–0.77, *p* = 0.010), and fulfilment of a LARC method (aOR = 0.14, CI: 0.04–0.48, *p* = 0.004). In contrast, there was no association with fulfilment of SARC (aOR = 0.46, CI: 0.15–1.40, *p* = 0.157) or coital methods (aOR = 1.50, CI: 0.74–3.06, *p* = 0.240).

**Table 2.** Conditional logistic models, among Wisconsin sexual and reproductive health patients not trying to be pregnant at any time point, 2020–2023

	Use of the preferred method						Method satisfaction					
	*OR*	*95% CI*	*p-value*	*aOR*	*95% CI*	*p-value*	*OR*	*95% CI*	*p-value*	*aOR*	*95% CI*	*p-value*
**Trouble getting wanted contraceptive method**												
No	1.00			1.00			1.00			1.00		
Yes	0.36	(0.20, 0.65)	0.002	0.36	(0.20, 0.64)	0.002	0.37	(0.18, 0.78)	0.012	0.39	(0.20, 0.77)	0.010
**Trouble due to availability^2^**												
No	1.00			1.00			1.00			1.00		
Yes	0.54	(0.27, 1.07)	0.072	0.50	(0.25, 1.01)	0.052	0.56	(0.23, 1.35)	0.178	0.66	(0.30, 1.49)	0.296
**Trouble due to affordability^3^**												
No	1.00			1.00			1.00			1.00		
Yes	0.50	(0.22, 1.14)	0.092	0.53	(0.21, 1.37)	0.176	0.47	(0.19, 1.12)	0.083	0.43	(0.18, 1.03)	0.058
**Missed healthcare visit**												
No	1.00			1.00			1.00			1.00		
Yes	0.65	(0.44, 0.96)	0.031	0.64	(0.43, 0.94)	0.026	0.58	(0.32, 1.04)	0.066	0.57	(0.32, 1.03)	0.059

Fulfillment^1^ of the LARC method						Fulfilment of the SARC method						Fulfilment of the coital method					
*OR*	*95% CI*	*p-value*	*aOR*	*95% CI*	*p-value*	*OR*	*95% CI*	*p-value*	*aOR*	*95% CI*	*p-value*	*OR*	*95% CI*	*p-value*	*aOR*	*95% CI*	*p-value*
**Trouble getting wanted contraceptive method**																	
1.00			1.00			1.00			1.00			1.00			1.00		
0.24	(0.08, 0.75)	0.018	0.14	(0.04, 0.48)	0.004	0.47	(0.15, 1.44)	0.169	0.46	(0.15, 1.40)	0.157	1.48	(0.73, 3.04)	0.254	1.50	(0.74, 3.06)	0.240
**Trouble due to availability^2^**																	
1.00			1.00			1.00			1.00			1.00			1.00		
0.47	(0.10, 2.17)	0.308	0.31	(0.07, 1.29)	0.099	0.64	(0.24, 1.69)	0.345	0.58	(0.23, 1.51)	0.245	1.23	(0.36, 4.19)	0.725	1.27	(0.38, 4.29)	0.675
**Trouble due to affordability^3^**																	
1.00			1.00			1.00			1.00			1.00			1.00		
0.09	(0.01, 0.65)	0.020	0.09	(0.01, 0.85)	0.037	0.53	(0.14, 2.04)	0.329	0.55	(0.13, 2.25)	0.375	1.87	(0.56, 6.26)	0.286	1.85	(0.54, 6.27)	0.297
**Missed healthcare visit**																	
1.00			1.00			1.00			1.00			1.00			1.00		
0.25	(0.08, 0.77)	0.020	0.24	(0.07, 0.80)	0.023	0.67	(0.27, 1.62)	0.345	0.66	(0.27, 1.64)	0.342	1.06	(0.48, 2.31)	0.884	1.06	(0.48, 2.35)	0.876

Notes: Covariates for adjusted models include: relationship status, insurance coverage, employment, and residence type. Fulfillment-adjusted models include employment only. ^1^Fulfillment refers to using the method type, among respondents who report a preference for the method type. Adjusted models include insurance coverage. ^2^Availability includes reasons stated as "it was too difficult to get to (no transportation or childcare, couldn't take time off work)", "the method that I wanted was not available at my doctor's office, clinic or pharmacy", "my source of health care is religiously affiliated, and thus does not provide the birth control method I wanted", "the doctor's office, clinic or pharmacy wasn't open when I could get there", "the place I usually go wasn't offering the care I needed because of the COVID-19 pandemic" (only included as a response option at baseline and biannuals 2 and 3), and write-in responses indicating scheduling availability challenges. ^3^Affordability includes reasons stated as "I couldn't afford it", "I didn't have insurance", "my health insurance doesn't cover it", and "the insurance co-pays/deductibles were too high".

When separating out different reasons for experiencing trouble getting contraceptive methods, experiencing trouble due to affordability was associated with lower odds of fulfilment of a LARC method (aOR = 0.09, CI: 0.01–0.85, *p* = 0.037) but not with current use of a preferred method (aOR = 0.53, CI: 0.21–1.37, *p* = 0.176), method satisfaction (aOR = 0.43, CI: 0.18–1.03, *p* = 0.058), or fulfilment by SARC (aOR = 0.55, CI: 0.13–2.25, *p* = 0.375) or coital method types (aOR = 1.85, CI: 0.54–6.27, *p* = 0.297). There were also no associations between experiencing difficulties due to availability and use of a preferred method (aOR = 0.50, CI: 0.25–1.01, *p* = 0.052), method satisfaction (aOR = 0.66, CI: 0.30–1.49, *p* = 0.296), fulfilment of LARC methods (aOR = 0.32, CI: 0.07–1.29, *p* = 0.099), fulfilment of SARC methods (aOR = 0.58, CI: 0.23–1.51, *p* = 0.245), and fulfilment of coital methods (aOR = 1.27, CI: 0.38, 4.29, *p* = 0.675). Missing a healthcare visit was associated with lower odds of currently using a preferred method (aOR = 0.64, CI: 0.43–0.94, *p* = 0.026) and fulfilment of LARC methods (aOR = 0.24, CI: 0.07–0.80, *p* = 0.023). Missing a healthcare visit was not associated with method satisfaction (aOR = 0.57, CI: 0.32–1.03, *p* = 0.059), fulfilment of SARC methods (aOR = 0.66, CI: 0.27–1.64, *p* = 0.342), nor fulfilment of coital methods (aOR = 1.06, CI: 0.48–2.35, *p* = 0.876).

When stratifying by SGM status, multiple associations emerged between barriers to care and contraceptive method use outcomes for transgender and/or queer respondents but not for cisgender and heterosexual respondents, with the exception of method satisfaction (Table 3). For cisgender and heterosexual respondents, experience of a missed wanted healthcare visit was associated with lower odds of method satisfaction (aOR = 0.48, CI: 0.25–0.92, *p* = 0.029). For transgender and/or queer respondents, experiencing trouble getting a wanted contraceptive method was associated with lower odds of current use of a preferred method (aOR = 0.18, CI: 0.08–0.42, *p* = 0.001) and satisfaction with the current method (aOR = 0.30, CI: 0.11–0.81, *p* = 0.022). When disaggregating specific reasons for trouble getting wanted contraceptive methods, trouble due to affordability only was associated with lower odds of preferred method use only (aOR = 0.18, CI: 0.05–0.68, *p* = 0.015). Trouble due to availability was not associated with either use of a preferred method (aOR = 0.47, CI: 0.20–1.09, *p* = 0.074) or method satisfaction (aOR = 0.44, CI: 0.13–1.45, *p* = 0.162). Lastly, missing a healthcare visit was associated with lower odds of current use of a preferred method (aOR = 0.37, CI: 0.20–0.67, *p* = 0.003) but not with method satisfaction (aOR = 0.62, CI: 0.24–1.61, *p* = 0.300) among transgender and/or queer respondents.

**Table 3.** Conditional logistic models, among Wisconsin sexual and reproductive health patients not trying to be pregnant at any time point, 2020–2023

	(A) Cisgender and heterosexual respondents											
	Use of the preferred method						Method satisfaction					
	*OR*	*95% CI*	*p-value*	*aOR*	*95% CI*	*p-value*	*OR*	*95% CI*	*p-value*	*aOR*	*95% CI*	*p-value*
**Trouble getting wanted contraceptive method**												
No	1.00			1.00			1.00			1.00		
Yes	0.63	(0.32, 1.25)	0.170	0.64	(0.32, 1.29)	0.192	0.49	(0.19, 1.22)	0.115	0.51	(0.21, 1.24)	0.127
**Trouble due to availability^1^**												
No	1.00			1.00			1.00			1.00		
Yes	0.61	(0.25, 1.49)	0.258	0.59	(0.24, 1.42)	0.220	0.89	(0.26, 3.01)	0.836	0.89	(0.26, 3.03)	0.842
**Trouble due to affordability**^2^												
No	1.00			1.00			1.00			1.00		
Yes	1.42	(0.47, 4.29)	0.508	1.55	(0.47, 5.08)	0.443	0.45	(0.12, 1.65)	0.206	0.51	(0.14, 1.87)	0.286
**Missed healthcare visit**												
No	1.00			1.00			1.00			1.00		
Yes	0.94	(0.57, 1.55)	0.791	0.93	(0.57, 1.51)	0.744	0.56	(0.28, 1.11)	0.089	0.48	(0.25, 0.92)	0.029

(B) Transgender and/or Queer respondents											
Use of the preferred method						Method satisfaction					
*OR*	*95% CI*	*p-value*	*aOR*	*95% CI*	*p-value*	*OR*	*95% CI*	*p-value*	*aOR*	*95% CI*	*p-value*
**Trouble getting wanted contraceptive method**											
1.00			1.00			1.00			1.00		
0.18	(0.07, 0.44)	0.001	0.18	(0.08, 0.42)	0.001	0.28	(0.10, 0.80)	0.021	0.30	(0.11, 0.81)	0.022
**Trouble due to availability^1^**											
1.00			1.00			1.00			1.00		
0.48	(0.21, 1.09)	0.075	0.47	(0.20, 1.09)	0.074	0.39	(0.11, 1.34)	0.124	0.44	(0.13, 1.45)	0.162
**Trouble due to affordability**^2^											
1.00			1.00			1.00			1.00		
0.19	(0.04, 0.86)	0.034	0.18	(0.05, 0.68)	0.015	0.48	(0.15, 1.62)	0.218	0.46	(0.14, 1.47)	0.174
**Missed healthcare visit**											
1.00			1.00			1.00			1.00		
0.42	(0.25, 0.73)	0.004	0.37	(0.20, 0.67)	0.003	0.61	(0.25, 1.50)	0.257	0.62	(0.24, 1.61)	0.300

Notes: Covariates for adjusted models include: relationship status, employment, and residence type. ^1^Availability includes reasons stated as "it was too difficult to get to (no transportation or childcare, couldn't take time off work)", "the method that I wanted was not available at my doctor's office, clinic or pharmacy", "my source of health care is religiously affiliated, and thus does not provide the birth control method I wanted", "the doctor's office, clinic or pharmacy wasn't open when I could get there", "the place I usually go wasn't offering the care I needed because of the COVID-19 pandemic" (only included as a response option at baseline and biannuals 2 and 3), and write-in responses indicating scheduling availability challenges. ^2^Affordability includes reasons stated as "I couldn't afford it", "I didn't have insurance", "my health insurance doesn't cover it", "the insurance co-pays/deductibles were too high".

## Discussion

We present and discuss three key takeaways from our study, illuminating the process of seeking contraceptive care and identifying ways in which it may be particularly challenging for SGM individuals.

First, our model results highlight how experiencing barriers to wanted contraception and needed healthcare is directly linked to diminished person-centred SRH outcomes. Reported experience of trouble getting contraceptive methods and missed health care visits are related to lower odds of using a preferred method and specifically for those wanting to use a LARC method. Experiencing any trouble getting contraception is also linked to lower method satisfaction. We see this relationship demonstrated as reports of experiencing barriers decrease over time, we see increases in satisfaction, use of a preferred method, and fulfilment of most method types, except for coital methods. These overall findings among SRH care seekers in Wisconsin are consistent with similar evidence documented among SRH patients in Arizona over time^26^ and corroborate existing cross-sectional evidence that highlights the connection between barriers to accessing SRH care and a variety of person-centred outcomes.^8,27–30^

Second, our study teases out how different types of access barriers, specifically availability and affordability, operate to impede fulfilment of specific preferred method types. When we focus on different types of access barriers in the overall study population, we find affordability of methods – not their availability – to be the barrier more closely linked to lower odds of fulfilled LARC use. This is especially noteworthy in our study sample, where there is a high level of insurance coverage, and respondents were all recruited as SRH patients in facilities that receive public funds to provide this care. This finding supports that cost barriers extend beyond insurance coverage, as other forms of financial and resource strains have been linked to reduced person-centred contraceptive outcomes.^6,28^ We know that patients often must employ multiple strategies to navigate the process of paying for care and, at times, negotiate whether to seek care at all.^30^ Additionally, our finding of an association between missed desired healthcare visits and lower levels of preferred contraceptive use, specifically for respondents who would prefer to be using LARC methods but not for respondents who would prefer to be using SARC or coital methods, further provides evidence that there may be unique or added barriers for potential users who desire methods that require engagement with a healthcare provider. LARCs, namely IUDs and implants, require a provider visit for initial insertion, which may have both direct (e.g. money) and indirect (e.g. time) costs. Most coital and many SARC methods, on the other hand, are increasingly available through more diverse non-provider-involved avenues, including via pharmacies and telehealth options.^31^

Third, we find the relationships described above between access barriers and person-centred outcomes over time to be especially robust for individuals in the SGM community, with this group reporting both different preferences for contraceptive method types and different barriers to realising these preferences from their cisgender heterosexual counterparts. Both affordability barriers, as well as missed needed healthcare, play a role in hindering their person-centred contraceptive outcomes. In contrast, only missed healthcare as a barrier was associated with lower levels of method satisfaction for cisgender and heterosexual respondents. Cost barriers to contraceptive access have been well-documented among various population groups,^5,6,30,32^ including SGM individuals.^16,33^ Although respondents across both sub-groups reported comparable fluctuations in preferred method usage, including fulfilment of permanent methods, SGM respondents consistently reported lower levels of both these person-centred outcomes while simultaneously reporting higher levels of missing needed healthcare, compared to their cisgender and heterosexual counterparts. These results may indicate that SGM individuals are less able to overcome the unique barriers to permanent methods and may be particularly motivated to access LARC methods.

Importantly, we note that the strong association documented between access barriers and subsequent person-centred outcomes for SGM individuals may be reflecting different underlying mechanisms for people who identify as any specific combination of sexual and gender minority identities. While individuals holding one or more of these identities share some common experiences of structural barriers and discrimination, such as a lack of provider training on LGBTQ + SRH competency, assumptions about contraceptive motivations, and poorer healthcare experiences due to their SGM status,^15,34^ there are also important needs and experiences that differ between these identities. For example, individuals who hold one or more gender minority identities may experience gender dysphoria in healthcare settings that use language to refer to “women’s” healthcare, pregnancy, and body parts that do not align with the individuals’ own language, or have providers who are not trained to understand how gender-affirming hormones and contraception can be used simultaneously, and require pelvic exams prior to obtaining some contraceptive methods.^35^ Individuals who hold one or more sexual minority identities may face provider assumptions about sexual partners and sexual activity, which influences which methods and related SRH care are recommended and provided to them.^36^ Additionally, the diverse set of gender and sexual identities and their intersections, as well as their sexual partners’ identities, can lead to a variety of unique needs and discrimination in healthcare settings.^35^

Specifically in Wisconsin, these findings may indicate that SGM individuals are more constrained than cisgender and heterosexual individuals despite local availability and prevalence of permanent contraceptive methods. According to the 2022 Wisconsin BRFSS report, Wisconsin has exceptionally high levels of vasectomy use and higher levels of IUD rates compared with other states.^37^ However, this finding may also reflect broader provider-level resistance to providing permanent contraception, especially for younger and nulliparous individuals, which may disproportionately impact SGM permanent contraception seekers.^38^ Although there is no statistical difference in age, the age distribution of SGM SRH care patients in our sample skewed younger than the cisgender and heterosexual users, and some SGM populations have a significantly lower prevalence of biological fertility intentions compared with the general population, which naturally plays into their contraceptive method preferences.^39^

These findings of differential experiences and outcomes for SGM individuals have clear implications for sexual and reproductive health equity.^‡‡^ Historically, individuals in the SGM community have reported lower quality of health care provider interactions in the context of SRH care compared to other groups^34,41^ and reported lower preferences for obtaining contraception in-person from a healthcare provider.^42^ This evidence, combined with national evidence underscoring lower quality of contraceptive care through telehealth services compared to in-person services,^43^ emphasises the importance of addressing disparities in care across all modalities of care and for all patients.^37^

Notably, this study precedes the 2022 *Dobbs v. Jackson Women’s Health Organization* SCOTUS decision, which eliminated the federal right to abortion care. Since that ruling, there has been a growing body of evidence documenting the broad impacts of subsequent state-level abortion bans or restrictions on the provision and accessibility of contraceptive and other SRH care. Since Wisconsin was one of the states in which abortion care was not available for many months following the *Dobbs* ruling, and Wisconsin reproductive-aged patients were already reporting barriers in accessing their preferred methods, it is possible that the strong link between access barriers and person-centred SRH outcomes documented in this study has increased, especially for those in marginalised communities.

Strengths of our analysis include our use of a panel dataset of a large sample of Wisconsin patients initially seeking SRH care at publicly supported healthcare facilities that is weighted to reflect the full patient population in the state, as well as our use of several patient-centred contraceptive method use outcomes.

We note some limitations of our study. First, the overlap of timing of our study period with two notable events that disrupted and shifted access to SRH care – the 2019 Title X Final Rule and the COVID-19 pandemic – make interpretation of the root causes for patients’ experiences of disruptions and/or barriers to care during this timeframe challenging. The announcement of the former event initialised increased contraceptive service utilisation, including LARC method uptake, in anticipation of potential disruptions.^44^ The latter event also ushered in increased telehealth opportunities for accessing contraception,^45^ which would have implications for respondents’ ability to access SARC methods during this study and may have played a role in the high reports of SARC and coital method fulfilment observed in our study.

Next, our sample consists of patients who reported high levels of insurance coverage and were already accessing SRH care at publicly funded healthcare facilities, many of which offer low or no-cost services to lower-income patients. This may have diluted the findings observed for the relationship between affordability access barriers and many of the person-centred outcomes examined, such that the impact of affordability barriers may be even more severe than we observed in our results for a less insured patient population or the general population.

Additionally, our composite SGM variable flattens the important distinctions regarding the SRH care needs and experiences of sexual minority and gender minority individuals. As noted above, evidence highlights that these two different aspects of individuals’ identity^46^ lead to distinct SRH needs and experiences that should be explored further with data able to tease out these differences.^47^ Although we believe that respondents who reported changes in sexual and gender identity throughout the study period may have experiences closer to respondents who identify as SGM at all time points, the addition of these respondents to the SGM stratification may potentially add to the flattening of the needs and experiences of sexual minority and gender minority individuals. As we may be examining a subpopulation whose needs are particularly in flux, this may overstate or understate differences by experience of barriers, depending on the unique composition of these respondents and what challenges or privileges they may hold when interacting with different aspects of contraceptive care.

Finally, this analysis focuses on examining the availability and affordability of contraceptive care access, just two of the multidimensional aspects of access to care. Acceptability of care^4^ also plays a key role in patient-centred outcomes^10,48^ and acceptability may be particularly important to assess for SGM individuals who have historically experienced higher levels of discrimination and bias in the healthcare setting.^34,36,41^ Future research should include a focus on understanding the interconnectedness amongst all dimensions of patient-centred healthcare access, and how barriers to any one of the dimensions might magnify other ones within the whole, especially for individuals who hold one or more marginalised identities.

## Conclusion

Findings from this study highlight the importance of continuing to rethink and rebuild systems of contraceptive care delivery to centre the needs of individuals like those in the SGM community who have historically faced increased barriers to accessing this care. There is a striking contrast in fulfilment of preferred contraception between transgender and/or queer respondents and cisgender and heterosexual respondents, and affordability and availability of access to contraceptive care seem to play a role. There is mounting evidence that these dimensions of access can be severely impacted by disruptions to care systems, such as shifting public funds and restrictions on any type of SRH access, including abortion. In pursuit of sexual and reproductive health equity, it is pertinent that policymakers safeguard what protections still exist and expand protective policies at both the state and federal levels that support access for SGM individuals.

## Author contributions

Conceptualisation: EL, CEG, FW, and MLK. Data curation: EL and CEG. Formal analysis: EL and CEG. Funding acquisition: MLK. Investigation: EL and CEG. Methodology: EL, CEG, and MLK. Project administration: EL and MLK. Software: EL and CEG. Supervision: EL and MLK. Validation: EL and CEG. Visualisation: CEG. Writing – original draft: EL, CEG, FW, and MLK.

## Footnotes

* The acronym LGBT/LGBTQ/LGBTQ+ is used in the studies referenced. However, in this paper, we refer to individuals who may identify as any gender or sexual orientation outside of cisgender or heterosexual as sexual and gender minorities.

† Baseline fielding for Wisconsin sites began in February 2020 until June 2021. Data collection paused from 30 March 2020–31 July 2020, due to the COVID-19 pandemic.

‡ The focus of the original RHIS study was on contraceptive care and given that most contraceptive methods are primarily used by individuals assigned female at birth, both the original study and this analysis focuses on the experiences of this population. Respondents are also able to report partner use of condom and vasectomy.

§ Respondents were asked “What sex were you assigned at birth, for example on your original birth certificate?” and were able to select “female”, “male”, “other”, and “prefer not to answer”.

** We considered receipt of SRH care to include receiving any of the following: prescription for a birth control method, check-up or medical test related to birth control, or counselling or information about birth control.

†† We recognise that this item could be considered an affordability-related delay (e.g. indirect cost), but we have categorised it as an availability-related delay because we believe it speaks more closely to the broader need to have accommodations to get to care that is not readily available close by.

‡‡ The condition in which systems ensure that all people have the necessary resources to attain their highest level of sexual and reproductive health.^40^

## Appendix 1. Distribution of key variables at each time point among Wisconsin sexual and reproductive health patients ages 15+ not trying to become pregnant at any timepoint, 2020–2023.

**Table:**

	All respondents					Diff. over time within all respondents
	Baseline^a^	6 months	12 months	18 months	24 months	
	Feb 2020 – Jun 2021	Feb 2021 – Dec 2021	Aug 2021 – Jun 2022	Feb 2022 – Dec 2022	Aug 2022 – Jun 2023	
*Among respondents not trying to become pregnant (Ns)*	900	634	685	678	710	
**Trouble getting wanted contraceptive method**	12.55	9.58	10.99	10.55	6.49	0.071
**Trouble due to availability^2^**	4.72	5.23	5.62	5.88	3.70	0.650
**Trouble due to affordability^3^**	6.60	2.93	3.60	4.18	1.30	0.006
**Missed healthcare visit when needed care**	38.80	33.43	36.16	34.76	26.81	0.006
*Patient-centred contraceptive method use outcomes*						
**Currently using any method**	82.56	85.60	80.66	76.09	77.84	0.003
**Satisfied with current method**	83.81	88.70	87.25	87.85	89.14	0.209
**Currently using preferred method**	82.09	79.24	78.72	78.33	88.68	0.002
**Fulfillment^1^ of wanted contraception by type of method**						
*Among respondents who want to be using LARCs*	259	180	184	175	198	
LARCs	67.32	83.26	80.52	80.69	87.63	<0.001
*Among respondents who want to be using SARCs*	499	317	339	313	284	
SARCs	86.07	88.57	86.55	82.63	89.62	0.242
*Among respondents who want to be using permanent methods*	102	73	79	85	86	
Permanent methods	30.37	30.52	33.32	28.54	52.38	0.042
*Among respondents who want to be using coital methods*	564	364	390	385	389	
Coital methods	92.14	86.51	83.54	81.08	87.41	0.008

**Table:**

	Cisgender and heterosexual respondents					Diff. over time within cisgender and hetero-sexual respond-ents
	Baseline^a^	6 months	12 months	18 months	24 months	
	Feb 2020 – Jun 2021	Feb 2021 – Dec 2021	Aug 2021 – Jun 2022	Feb 2022 – Dec 2022	Aug 2022 – Jun 2023	
*Among respondents not trying to become pregnant (Ns)*	651	447	484	477	487	
**Trouble getting wanted contraceptive method**	11.37	8.22	9.93	8.97	5.13	0.171
**Trouble due to availability^2^**	4.13	2.96	4.18	5.33	2.07	0.444
**Trouble due to affordability^3^**	5.49	3.19	3.67	2.82	1.15	0.048
**Missed healthcare visit when needed care**	35.71	29.35	32.67	30.97	22.71	0.003
*Patient-centred contraceptive method use outcomes*						
**Currently using any method**	81.78	85.00	82.38	74.50	76.34	0.009
**Satisfied with current method**	84.67	89.15	86.61	87.99	89.20	0.515
**Currently using preferred method**	85.51	79.38	79.64	81.58	90.59	0.004
**Fulfillment^1^ of wanted contraception by type of method**						
*Among respondents who want to be using LARCs*	160	116	116	104	122	
LARCs	67.79	79.49	78.62	82.76	83.74	0.107
*Among respondents who want to be using SARCs*	388	243	264	231	211	
SARCs	87.71	88.83	86.44	83.58	91.94	0.252
*Among respondents who want to be using permanent methods*	58	45	48	52	50	
Permanent methods	48.93	32.45	34.81	31.20	56.65	0.113
*Among respondents who want to be using coital methods*	405	260	278	272	261	
Coital methods	92.54	85.53	84.58	78.49	88.33	0.003

**Table:**

	Transgender and/or queer respondents					Diff. over time within transgender and/or queer respondents
	Baseline^a^	6 months	12 months	18 months	24 months	
	Feb 2020 – Jun 2021	Feb 2021 – Dec 2021	Aug 2021 – Jun 2022	Feb 2022 – Dec 2022	Aug 2022 – Jun 2023	
*Among respondents not trying to become pregnant (Ns)*	239	182	196	193	215	
**Trouble getting wanted contraceptive method**	16.03	13.01	13.65	13.51	8.87	0.522
**Trouble due to availability^2^**	6.39	10.58	9.15	7.03	7.02	0.683
**Trouble due to affordability^3^**	9.68	2.43	3.47	6.89	1.15	0.052
**Missed healthcare visit when needed care**	47.80	42.69	44.19	41.81	36.59	0.449
*Patient-centred contraceptive method use outcomes*						
**Currently using any method**	85.89	86.90	76.98	81.19	80.83	0.195
**Satisfied with current method**	81.47	87.46	88.65	87.43	88.70	0.416
**Currently using preferred method**	73.71	78.48	76.23	72.09	84.71	0.224
**Fulfillment^1^ of wanted contraception by type of method**						
*Among respondents who want to be using LARCs*	96	64	65	68	75	
LARCs	65.79	88.72	82.98	77.89	92.67	0.002
*Among respondents who want to be using SARCs*	108	72	74	80	70	
SARCs	79.89	86.74	86.89	79.96	81.63	0.704
*Among respondents who want to be using permanent methods*	44	28	31	32	35	
Permanent methods	10.75	27.99	30.82	25.81	42.90	0.048
*Among respondents who want to be using coital methods*	155	104	110	111	123	
Coital methods	90.99	88.76	80.85	86.97	85.89	0.454

Notes: Sample represented in the table includes Wisconsin family planning patients ages 15 and older recruited between 2020 and 2021 and indicating that they were not trying to become pregnant at any timepoint during the study period. ^a^Baseline fielding paused between 20 March 2020 and 31 July 2020 due to the COVID-19 pandemic. ^1^Fulfillment is constructed as among respondents who want to use the preferred method type, those who report using it. ^2^Availability includes reasons stated as "it was too difficult to get to (no transportation or childcare, couldn't take time off work)", "the method that I wanted was not available at my doctor's office, clinic or pharmacy", "my source of health care is religiously affiliated, and thus does not provide the birth control method I wanted", "the doctor's office, clinic or pharmacy wasn't open when I could get there", "the place I usually go wasn't offering the care I needed because of the COVID-19 pandemic" (only included as a response option at baseline and biannuals 2 and 3), and write-in responses indicating scheduling availability challenges. ^3^Affordability includes reasons stated as "I couldn't afford it", "I didn't have insurance", "my health insurance doesn't cover it", "the insurance co-pays/deductibles were too high".
